# Advances in Induced Pluripotent Stem Cell Reprogramming and Its Application in Amyotrophic Lateral Sclerosis: A Review

**DOI:** 10.1096/fba.2025-00126

**Published:** 2025-10-31

**Authors:** Yingliu Luo, Zhenru Xu, Zunxiong Li

**Affiliations:** ^1^ Department of Clinical Laboratory The Second Affiliated Hospital of Hainan Medical University Haikou People's Republic of China; ^2^ Department of Dermatology The First Affiliated Hospital of Hainan Medical University Haikou People's Republic of China

**Keywords:** amyotrophic lateral sclerosis, induced pluripotent stem cell, motor neuron

## Abstract

Since Yamanaka's landmark achievement in reprogramming somatic cells into induced pluripotent stem cells (iPSCs) using the four key transcription factors—OCT4, SOX2, KLF4, and c‐Myc (OSKM)—iPSC technology has made significant strides. Notable advancements include refining reprogramming factors, delivery systems, somatic cell selection, and optimization of reprogramming conditions, along with developing chemical reprogramming methods. With their unparalleled proliferative capacity and near‐pluripotent differentiation potential, iPSCs have become invaluable tools for investigating neurodegenerative diseases, including amyotrophic lateral sclerosis (ALS). Neuronal models derived from ALS patient‐specific iPSCs, particularly iPSC‐derived motor neurons (iPSC‐MNs), offer a robust platform to recapitulate disease‐specific pathology and investigate the molecular mechanisms underpinning ALS, thereby accelerating the discovery of novel therapeutic strategies. This review highlights the evolution of iPSC technology and its transformative applications in ALS modeling, drug discovery, and therapeutic development.

## Graphical abstract

iPSC Development and iPSC Application in ALS.

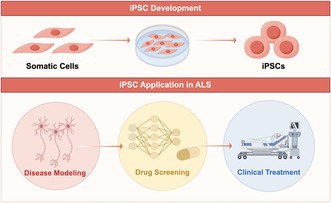

**Abbreviations**
4‐Oct: octamer‐binding transcription factor 4
8‐Br‐cAMP: 8‐Bromoadenosine 3′,5′‐cyclic monophosphate
ALS: amyotrophic lateral sclerosis
BDNF: brain‐derived neurotrophic factor
BMP: bone morphogenetic protein
CAR‐T: chimeric antigen receptor T‐cell
c‐Myc: cellular myelocytomatosis
CRISPR‐Cas: clustered regularly interspaced short palindromic repeats—associated protein
DMEM/F12: dulbecco's modified eagle medium/Ham's F12
DMH1: dorsomorphin homolog 1
DOT1L: disruptor of telomeric silencing 1‐like
EBV: Epstein–Barr virus
Esrrb: estrogen‐related receptor beta
GDNF: glial cell‐derived neurotrophic factor
Glis1: GLI‐similar zinc finger protein 1
iPSC: induced pluripotent stem cell
Klf4: Krüppel‐like factor 4
LIN28: lin‐28 homolog
L‐Myc: lung myelocytomatosis‐related protein
miRNA: micro RNA
mRNA: Messenger RNA
Nanog: homeobox transcription factor nanog
Nr5a2: nuclear receptor subfamily 5 group a member 2
NSC: neural stem cell
RA: retinoic acid
RepSox: a small molecule replacing Sox2 in reprogramming
SAG: smoothened agonist
SHH: sonic hedgehog
SMAD: suppressor of mothers against decapentaplegic
Sox2: SRY (sex determining region Y)‐Box 2
SUV39H1: suppressor of variegation 3‐9 homolog 1
TCR: T cell receptor
TGF‐β: transforming growth factor beta
VPA: valproic acid
WNT: wingless‐related integration site
YY1: Yin Yang 1 transcription factor

## 1 Introduction

The success of somatic cell nuclear transfer demonstrated that somatic cells carry the same genetic code as the zygote and that activating specific portions of this code is sufficient to reprogram cells to an early developmental state [1, 2]. Nearly half a century later, the discovery of induced pluripotent stem cells (iPSCs) provided a molecular mechanism underlying this reprogramming process. In 2006, Takahashi and Yamanaka's group used a silico subtraction experiment to confirm that overexpressing the four transcription factors, octamer‐binding transcription factor 4 (Oct4), SRY (Sex determining region Y)‐box2 (Sox2), Krüppel‐like factor 4 (Klf4), and cellular‐Myelocytomatosis (c‐Myc) in mouse fibroblasts could generate embryonic stem cell‐like cells, iPSCs. In the following year, they further demonstrated that the same four factors could reprogram human fibroblasts into human iPSCs. Thereafter, OCT4, SOX2, KLF4, and c‐Myc became known as the OSKM factors, which were key to somatic cell reprogramming into iPSCs [3, 4]. From that point forward, iPSC technology ushered in rapid development. The combination and optimization of multiple factors and vectors in somatic cell reprogramming have significantly streamlined the technical procedures and reduced the time required, while also expanding the pool of somatic cells eligible for reprogramming. With the development of iPSC technology and the elucidation of its molecular mechanisms, somatic cell reprogramming can now be achieved without exogenous gene expression, relying instead on small molecule combinations, known as chemical reprogramming, which significantly enhance the safety of iPSCs and their potential for clinical applications [5, 6]. During early‐phase human chemical reprogramming, a distinct highly plastic intermediate cell state emerges, exhibiting enhanced chromatin accessibility and activation of early embryonic developmental genes. Notably, comparative analyses with cellular dedifferentiation processes in lower organisms reveal that this stage activates gene expression signatures analogous to those observed during initial limb regeneration in axolotls [7]. In this review, we discuss recent advancements in somatic cell reprogramming to generate iPSCs, covering various methods such as reprogramming factors, delivery systems, culture conditions, somatic cell selection, and chemical induction. Additionally, we summarize the roles and potential of iPSC‐derived products in ALS disease modeling, drug screening, and clinical applications.

## 2 iPSC Reprogramming

### 2.1 The Reprogramming Factors

The identification and optimization of reprogramming factors are pivotal to advancing iPSC technology. A key aspect of this effort is identifying factors that minimize the risk of tumorigenesis and maximize reprogramming efficiency.

Studies have shown that c‐Myc, a reprogramming factor within the OSKM combination, acts as an oncogene, with its overexpression contributing to tumorigenesis. This poses significant risks to the stability and safety of iPSCs, making it urgent to identify factors that can reduce the use of c‐Myc or serve as alternatives [8, 9, 10]. In 2007, Takahashi and Yamanaka published a study demonstrating that somatic cell reprogramming into iPSCs could be achieved by expressing only the transcription factors OCT4, SOX2, and KLF4, without the need for c‐Myc, indicating that c‐Myc is not essential for reprogramming. Later, the authors discovered that substituting L‐Myc for c‐Myc reduced the tumorigenic risk of iPSCs while maintaining reprogramming efficiency [4, 10]. In the same year, another study by Thomson JA et al. showed that OCT4, SOX2, NANOG, and LIN28 (OSNL) are sufficient to reprogram human somatic cells to pluripotent stem cells, which addresses the potential tumorigenic risks of c‐Myc [11]. These findings suggest that reprogramming factors can be flexibly combined and that family members with similar functions can substitute for one another. Further research has demonstrated that KLF2 and KLF5 can substitute for KLF4, while SOX1 and SOX3 can replace SOX2, and L‐Myc and N‐Myc can substitute for c‐Myc. Notably, although some family members can substitute for their original OSKM counterparts, substantial differences in reprogramming efficiency persist, with most alternatives exhibiting significantly lower efficiency compared to the OSKM combination [10, 11, 12, 13]. Moreover, studies have shown that genes or small molecules not belonging to the reprogramming factor family can have similar effects. Heng JC et al. found that NR5A2 can substitute for OCT4 and, in combination with SOX2 and KLF4, induce somatic cell reprogramming into iPSCs. The small molecule RepSox can replace SOX2 in reprogramming when used with other factors. Huck‐Hui Ng et al. demonstrated that Esrrb and Glis1 can also serve as alternatives to c‐Myc in somatic cell reprogramming with comparable effectivity [14, 15, 16].

Building upon the optimization of safety, selecting factors to enhance iPSC generation efficiency remains a central focus of iPSC technology. Shortly after the establishment of iPSC technology, Tsubooka N et al. discovered that replacing c‐Myc with SALL4 could reprogram somatic cells into iPSCs, addressing the safety concerns associated with c‐Myc while preserving the reprogramming effect [17]. Subsequently, Edel MJ et al. found that in the absence of c‐Myc, factors such as Rem2, cyclin D1, and p53 inhibition were also efficient in reprogramming somatic cells into iPSCs [18]. The combination of p53 inhibition with different reprogramming factors markedly increased iPSC generation efficiency [19]. Moreover, miRNAs and genes regulating miRNA biogenesis, such as miR‐302/367, miR‐372, and Lin28, notably improve the reprogramming of somatic cells to pluripotency [20, 21]. Chromatin remodeling genes, including SUV39H1, YY1, DOT1L, and Jhdm1a/1b, as well as epigenetic modulators such as DNA methyltransferase inhibitors (5‐aza‐cytidine, RG108), histone deacetylase inhibitors (Sodium butyrate, Trichostatin A), and the histone methylation regulator Neplanocin A (DZNep), have been shown to enhance the robustness of reprogramming [5, 22, 23, 24, 25, 26]. Additionally, Ying Wang et al. discovered that with 8‐Bromoadenosine 3′, 5′‐cyclic monophosphate (8‐Br‐cAMP), human fibroblast reprogramming into iPSCs was improved by twofold. Furthermore, combining 8‐Br‐cAMP with valproic acid (VPA) increased iPSC generation efficiency by up to 6.5‐fold [27, 28]. Meanwhile, we came across an intriguing report demonstrating that expressing OCT4 alone in human neural stem cells can successfully generate iPSCs, which highlights the pivotal role of OCT4 in the reprogramming system [29]. As for the combination of reprogramming factors, it is essential to consider the individual factors throughout the reprogramming process, as the types and numbers of factors required may differ across cell types.

### 2.2 Delivery Systems for Reprogramming

Table 1 summarizes the characteristics of various vector/platform types, including core attributes such as vector type (Virus/DNA/RNA/Protein), genetic material, genomic integration capability, and comparative data across platforms including retrovirus/lentivirus, Sendai virus (SeV), adenovirus, plasmid, episomal plasmid, PiggyBac transposon, minicircle DNA, synthetic RNA, and recombinant protein.

**TABLE 1:** Different reprogramming methods.

Property	Retro/lentivirus	Sendai virus (SeV)	Adenovirus	Plasmid	Episomal plasmid	PiggyBac transposon	Minicircle DNA	SyntheticRNA	Recombinant protein
Vector/platform type	Virus	Virus	Virus	DNA	DNA	DNA	DNA	RNA	Protein
Genetic material	RNA	RNA	dsDNA	DNA	DNA	DNA	DNA	RNA	None
Genomic integration capacity	Yes (random integration)	No (cytoplasmic RNA virus)	No (episomal)	Low (fortuitous)	No (episomal)	Yes (site‐specific integration)	No (episomal)	No (cytoplasmic)	No
Site of action	Nucleus	Cytoplasm	Nucleus	Nucleus	Nucleus	Nucleus	Nucleus	Cytoplasm	Cytoplasm /Nucleus
Requirement for viral enzymes	Yes (reverse transcriptase, integrase)	No	No	No	No	No (requires transposase)	No	No	No
Ability to infect or act upon non‐dividing cells	Yes	Yes	Yes	No	No	No	No	Yes (requires delivery tool, e.g., electroporation)	Yes (requires delivery tool)
Possibility of transgene excision	Difficult/ Impossible	Easy	Easy	Difficult	Easy (diluted over time)	Possible (transposase expression)	Easy	Easy	Easy
Reprogramming efficiency	High (+++)	High (+++)	Low (+)	Low (+)	Moderate (++)	Moderate (++)	Low to Moderate (+/++)	High (+++)	Low (+)
Approximate reprogramming time	2‐3 weeks	3‐4 weeks	3‐4 weeks	4‐5 weeks	3‐4 weeks	3‐4 weeks	3‐4 weeks	2–3 weeks	4‐6 weeks
Mode of application	Ex vivo	Ex vivo	In vivo	Ex vivo	Ex vivo	Ex vivo	In vivo	In vivo	Ex vivo

#### 2.2.1 Integrative Viral Vectors

Unlike the direct replication strategy employed by typical RNA viruses, retroviruses propagate through “RNA–DNA–RNA” pathway: Viral RNA undergoes reverse transcription into proviral DNA catalyzed by reverse transcriptase, integrates into the host genome, and subsequently exploits the host transcriptional machinery to produce progeny viral RNA and structural proteins, culminating in the assembly and release of nascent virions. Retroviruses can stably integrate into the host genome, establishing their position as one of the most widely used viral vectors for stable gene expression in mammalian cells, backed by decades of successful applications. The first somatic cell reprogramming into iPSCs relied specifically on viral vectors, including retroviruses and lentiviruses. These vectors facilitated the stable expression of key transcription factors in host cells. In 2007, Yamanaka et al. reprogrammed human cells into iPSCs using retroviral vectors to introduce OCT4, SOX2, KLF4, and c‐Myc [4], while Thomson JA et al. used lentiviral vectors to deliver OCT4, SOX2, Nanog, and LIN28 to achieve similar results [11]. The use of viral vectors was critical to the high efficiency of early iPSC generation and laid the foundation for iPSC technology. Thereafter, techniques employing viral vectors to reprogram various somatic cells into iPSCs were gradually developed. For instance, in 2008, Trond Aasen et al. successfully reprogrammed keratinocytes into iPSCs by introducing OSKM through retroviral vectors [30]. Although viral vectors exhibit relatively high reprogramming efficiencies (0.1%–1%), their genome integration risks, which result in unpredictable genetic mutations and tumorigenesis, severely limit their potential for clinical translation. Studies have shown that retroviruses used to deliver reprogramming factors may lead to multicopy chromosomal integration in the iPSC genome. This increases the risk of genomic toxicity, which not only contributes to tumor formation in chimeric mice generated with iPSCs but also compromises the maintenance of iPSC pluripotency [31, 32, 33].

#### 2.2.2 Non‐Integrative Viral Vectors

##### 2.2.2.1 Adenovirus

The adenoviral genome is a linear double‐stranded DNA molecule. After infecting the host cell, the viral DNA is translated into viral proteins and then assembled with newly replicated viral DNA into infectious virions, which are subsequently released from the cell. Since the adenoviral genome differs from the human genome in lacking the specific sequences and enzymes necessary for integration into the host genome, adenoviral vector plasmids are theoretically free of genomic integration risks and are widely recognized as one of the most used vectors in iPSC technology [34, 35, 36]. As early as 2008, Stadtfeld M et al. successfully reprogrammed mouse fibroblasts and hepatocytes into iPSCs using adenoviral vector plasmids. Subsequent evaluations of pluripotency and safety demonstrated that these adenovirus‐induced iPSCs exhibited characteristics and differentiation potential comparable to embryonic stem cells. Further teratoma formation assays confirmed the presence of distinct tri‐lineage tissues, and no evidence of viral genomic integration was observed in the generated iPSCs [34]. In 2009, Zhou W et al. reprogrammed human embryonic fibroblasts into iPSCs using adenoviral vectors to deliver OSKM factors. Similarly, no viral genomic integration was observed in the resulting iPSCs [37]. These studies suggest that adenoviral vectors are a relatively safe reprogramming method for generating iPSCs. However, they also have certain limitations, the most significant being their relatively low reprogramming efficiency. Adenoviral vector‐mediated reprogramming shows extremely low efficiency, with rates of 0.001%–0.0001% in mouse somatic cells and 0.0002% in human somatic cells, significantly below those of lentiviral and retroviral methods. Improving reprogramming efficiency is key to advancing the role of adenoviral vectors in iPSC technology [34, 37].

##### 2.2.2.2 Sendai Virus

Sendai virus, a single‐stranded RNA virus, remains in the cytoplasm during infection, eliminating the risk of nuclear integration into the host genome. The target genes delivered by Sendai viral vectors can stably and continuously express in the cytoplasm, making it a commonly used tool in basic and applied biological research [38]. Studies have shown that Sendai viral vectors can safely and effectively reprogram somatic cells, such as fibroblasts and T cells, into iPSCs, with reprogramming efficiencies of up to 1% for fibroblasts and 0.1% for T cells, making them a widely used tool in iPSC generation [39, 40]. However, compared to adenoviral vectors, Sendai viral vectors are more refractory to elimination. Complete clearance typically necessitates over 10 cell passages or culturing cells at 39°C, introducing additional technical challenges.

##### 2.2.2.3 PiggyBac Transposon

The PiggyBac transposon is a mobile DNA element originally isolated from the cabbage looper moth, *Trichoplusia ni* , in the 1980s. It contains inverted terminal repeats (ITRs) at both ends, which are specifically recognized and cleaved by the PiggyBac transposase. Upon excision, the transposon preferentially integrates into TTAA sites within the host genome. When the PiggyBac transposase is re‐expressed, it facilitates precise excision of the transposon, restoring the original TTAA sequence at the insertion site. This dual capability for accurate integration and excision makes PiggyBac a powerful tool for gene engineering and reprogramming technologies in mammalian cells [41]. In 2009, Kaji et al. were the first to employ the PiggyBac transposon to deliver reprogramming factors into somatic cells, successfully generating iPSCs. This approach offers an efficient and relatively safe method for iPSC generation, as the PiggyBac transposon can be precisely excised from the host genome following reprogramming, yielding footprint‐free iPSCs [42, 43, 44]. However, studies have indicated that while the PiggyBac transposon can be excised from the genome, it may unpredictably reactivate and reintegrate into off‐target sites, resulting in genomic instability. Moreover, the need for precise control of transposase expression and transposon excision adds to the complexity of experimental workflows [45].

#### 2.2.3 Non‐Integrative Non‐Viral Vectors

##### 2.2.3.1 mRNA

The introduction of messenger RNA (mRNA) encoding reprogramming factors offers a non‐integrative approach for the rapid, efficient, and safe generation of iPSCs [46]. In 2010, Warren L et al. demonstrated that synthetically modified mRNA encoding reprogramming factors (OSKML: OCT4, SOX2, KLF4, c‐Myc, Lin28) could efficiently reprogram human fibroblasts into iPSCs within 20 days. They also found that the addition of valproic acid (VPA) during reprogramming significantly enhanced iPSC generation efficiency from 1.4% to 4.4%. mRNA reprogramming circumvents the risks of genomic integration, thereby reducing the potential for genetic mutations and oncogenesis. However, it has certain limitations, including poor mRNA stability, high susceptibility to degradation by intracellular nucleases, and the need for frequent transfection to maintain factor expression. Despite these challenges, mRNA reprogramming remains an important method in iPSC generation research due to its notable safety and efficiency [46, 47, 48].

##### 2.2.3.2 Episomal Vector

Episomal vectors are considered a theoretically safe and simple method for iPSC generation. As early as 2008, Yamanaka revealed that transfection of DNA reprogramming factor plasmids into mouse fibroblasts could generate iPSCs, though with relatively low efficiency. Nevertheless, the success of this non‐viral, non‐integrating somatic cell reprogramming into iPSCs undoubtedly paved the way for the development of safer and more efficient DNA plasmid vectors for iPSC generation. In 2009, Thomson et al. further refined this approach by using oriP/EBNA1‐based non‐viral, non‐integrating episomal vectors to generate iPSCs [49, 50, 51]. oriP/EBNA1, derived from the Epstein–Barr virus (EBV), is a viral element that promotes the replication of episomal plasmid DNA in dividing cells, enabling the continued presence of exogenous reprogramming factors, which facilitates iPSC generation. Additionally, modifications to plasmid transfection methods, such as electroporation, nuclear transfection, and the use of various reagents (e.g., Lipo2000, Lipo3000), can further improve the efficiency of iPSC generation. Notably, LONAZ's nuclear transfection system, combined with episomal plasmids and its corresponding reagents, provides a novel and effective approach for the safe generation of iPSCs. The use of nuclear transfection technology and oriP/EBNA1‐based episomal plasmids, which do not integrate into the genome and are gradually lost during cell proliferation, provides a safer, simpler, and more efficient approach to somatic cell reprogramming, making it a promising candidate for clinical application.

### 2.3 Selection of Somatic Cells for Reprogramming

A variety of somatic cell types have been successfully reprogrammed into iPSCs, including fibroblasts [4, 10], urinary cells [52, 53], keratinocytes [54, 55], hepatocytes [56], renal epithelial cells [57], astrocytes [58], adipose stem cells [59, 60], neural stem cells [29, 61], amniotic fluid‐derived cells [62, 63], human umbilical vein endothelial cells [64], and various human peripheral blood cells [65, 66]. Advancements in iPSC technology, particularly optimizing reprogramming vectors and improving efficiency, have expanded the range of somatic cells available for reprogramming. Currently, peripheral blood‐derived mononuclear cells, including T cells, B cells, and CD34+ hematopoietic stem cells, are increasingly considered an ideal choice for reprogramming [67, 68, 69]. Compared to invasive methods requiring surgical procedures to obtain fibroblasts or mesenchymal stem cells, peripheral blood mononuclear cells provide a more accessible and less intrusive source. They have minimal impact on the donor and are well‐accepted by patients, making them ideal for the generation of disease‐specific iPSC banks and their clinical applications. Furthermore, numerous studies have demonstrated that iPSCs reprogrammed from peripheral blood‐derived T cells resemble embryonic stem cells more closely and differentiate more readily into T cells, thereby enhancing their potential for tumor therapies (e.g., CAR‐T). The distinct feature of TCR rearrangement enables the labeling and differentiation of iPSC‐derived T cells from host cells, with significant clinical implications [70, 71, 72].

### 2.4 Optimization of Reprogramming Conditions

The rapid advancement of iPSC technology is closely associated with continuously optimizing culture conditions, including refining iPSC culture media, using feeder cells, and developing extracellular matrix (ECM) components [73, 74]. Early iPSCs culture systems utilized serum‐containing components and mouse feeder cells for support. While effective, these conditions posed challenges in terms of standardization and batch‐to‐batch variability. The use of mouse feeder cells also heightened the risk of exogenous contamination and introduced variability in iPSC quality. The condition of feeder cells during reprogramming was critical to iPSC generation, adding complexity and instability to the experimental process. The development of iPSC culture media has progressed toward replacing serum with defined components. For example, in 2006, Yamanaka's team employed DMEM/F12 supplemented with KnockOut Serum Replacement, basic fibroblast growth factor (bFGF), insulin, and transferrin for iPSC cultivation. This was later followed by the introduction of fully defined, animal‐free media, such as mTeSR1 [4, 11, 59, 75, 76, 77]. mTeSR1 is a serum‐free culture medium containing human basic fibroblast growth factor (rh bFGF) and recombinant human transforming growth factor β (rh TGFβ)., which enhances both the stability and efficiency of iPSC culture. Stemcell Technologies has developed an upgraded version, mTeSR Plus, which optimizes critical components (including FGF2) and enhances pH buffering capacity. Both mTeSR1 and mTeSR Plus must be used in conjunction with 5X Supplement. When combined with extracellular matrices such as Matrigel or fibronectin (including Corning Matrigel hESC‐Qualified Matrix and Vitronectin XF), they effectively support the maintenance and proliferation of iPSCs. These media are also applicable for somatic cell reprogramming to generate iPSCs, thereby enhancing the quality and stability of iPSC generation while minimizing the risk of xenogenic contamination from feeder cells [78, 79, 80, 81, 82, 83]. Additionally, several animal‐free iPSC culture media have been developed, including TeSR‐E8, eTeSR, NutriStem hPSC XF Medium, and StemFit Basic03. These defined media have promoted standardization and improved reproducibility in iPSC culture and expansion, thereby enhancing the robustness and clinical applicability of iPSC technology and facilitating its application in clinical research [84, 85, 86].

### 2.5 Chemical Reprogramming for iPSC Generation

Deng H et al. developed a landmark technique for chemical reprogramming of iPSCs, utilizing a combination of small molecules to replace traditional exogenous factors in the reprogramming of somatic cells into iPSCs. In 2013, their team successfully reprogrammed mouse somatic cells into iPSCs using only exogenous chemical small molecules with molecular weights typically under 900 daltons (Da) or even at nanoscale dimensions. These compounds exhibit biological activity by directly targeting both intracellular and cell‐surface proteins that play pivotal roles in cellular signal transduction and epigenetic regulation. They possess distinct pharmacological advantages, including excellent membrane permeability, facile chemical synthesis, cost‐effectiveness, precise dose‐titration capability, and superior biological plasticity. After nearly a decade of research, they achieved the first successful chemical reprogramming of human cells in 2022 [5, 6]. Unlike traditional iPSC generation methods, such as somatic cell nuclear transfer or the introduction of transcription factors, chemical reprogramming relies solely on small molecule combinations, offering a more streamlined and adaptable approach. This technique allows for precise monitoring of cellular changes at each stage and provides enhanced spatiotemporal control, facilitating reversible modulation of the reprogramming process. As a non‐integrative method, it presents a safer and more controllable alternative to traditional gene‐based reprogramming techniques, reducing potential safety risks associated with genetic modification.

Chemical reprogramming is a staged process with precise regulation at each phase. Initially, human cells are induced into epithelial‐like cells using a combination of six small molecules: CHIR99021, 616452, TTNPB, Y27632, SAG, and ABT‐869 [6]. Tranylcypromine, 5‐Azacytidine [87], and JNKIN8 are first introduced to demethylate the epithelial‐like cells, maintaining their high proliferative capacity and plastic intermediate state. Subsequently, a combination of small molecules, including CHIR99021, 616452, Valproic acid, Tranylcypromine, DZNep, EPZ004777, UNC0379, Y27632, and PD0325901, activates OCT4 expression, thereby directing the cells toward an extraembryonic endoderm‐like (XEN‐like) stage. Finally, the plastic intermediate cells are induced into human pluripotent stem cells through a combination of CHIR99021, PD0325901, SB590885, IWP2, and Y27632. This approach not only establishes a novel reprogramming strategy but also elucidates cell fate determination mechanisms. Furthermore, it opens avenues for disease modeling and clinical translation, advancing stem cell applications in regenerative medicine.

### 2.6 Genomic Safety of Donor‐Derived iPSCs: From Biobanking to Clinical Translation

Donor somatic cell‐derived iPSCs carry the complete nuclear genome of the donor. These genetic variations are retained during the reprogramming process and become an inherent genetic background of the iPSC line [88]. The genetic load includes gene sequence variants classified as pathogenic or likely pathogenic by the American College of Medical Genetics and Genomics (ACMG), such as variations associated with Mendelian disorders (e.g., hypertrophic cardiomyopathy) or high‐penetrance cancer predisposition syndromes (e.g., *TP53* mutations). These variants may directly lead to functional abnormalities or malignant transformation of derived cells after transplantation [89]. Common polygenic disease risk alleles (e.g., gene loci associated with type II diabetes or Alzheimer's disease), while not directly pathogenic, may affect the long‐term functional stability of differentiated cells or their response to the micro‐environment [90]. Pharmacogenomic‐related variations may influence the cellular metabolic response to therapeutic drugs, leading to unpredictable toxicity in co‐administration scenarios [91]. Mitochondrial DNA (mtDNA) mutations accumulated in somatic cells are retained and may be amplified after reprogramming, not only affecting the cellular energy metabolism state but also potentially encoding abnormal proteins that act as neoantigens triggering immune recognition [92]. Additionally, polymorphisms in immune‐related genes may affect the immunocompatibility of transplanted cells. On the other hand, epigenetic memory causes iPSCs derived from donor cells through reprogramming to retain a differentiation preference for their original tissue lineage [93]. For example, iPSCs derived from blood cells may more easily differentiate toward hematopoietic directions, while those derived from fibroblasts may tend toward mesenchymal lineage differentiation. This memory may affect the efficiency, purity, and functional maturity when iPSCs are directionally differentiated into target cell types (such as neurons or cardiomyocytes), leading to heterogeneity and unpredictability in derived cells, thereby posing potential safety and efficacy risks [94]. The epigenetic memory of iPSCs may affect the function of terminal cells and is an important consideration in safety assessment.

Currently, multiple studies hold different views on whether new variations are introduced during iPSC reprogramming and culture. A 2016 study reported that iPSC cell lines gain an average of 0.8–1.2 SNV mutations per cell per passage, only one‐tenth the number of mutations gained per passage in somatic cells [94]. In 2017, researchers from the National Human Genome Research Institute (NHGRI), based on whole‐exome sequencing analysis, confirmed that most mutations in iPSCs come from rare genetic mutations in parental fibroblasts and confirmed that the cell reprogramming process does not increase the probability of genetic mutation occurrence [95]. This conclusion still requires cautious treatment. Studies have found that abnormal gene expression may still lead to immune rejection after autologous iPSC transplantation [95], and de novo mutations in mitochondria have been proposed as a potential source of neoantigens for autologous iPSCs [92]. However, current research debates the immunogenicity of iPSCs [96, 97]. Allogeneic iPSCs, while reducing costs and increasing accessibility, face more prominent immunogenicity issues. Traditional methods involve using immunosuppressants, while current research focuses on super‐donor stem cell banks and HLA cloaking techniques.

The construction strategy of allogeneic iPSC banks mainly focuses on reducing immune rejection risk through human leukocyte antigen (HLA) matching, by establishing “super‐donor” stem cell banks to cover a large number of recipient populations. HLA Cloaking. Knocking out the common component of class I major histocompatibility complex, the β2‐microglobulin (B2M) gene, or one of the four key transcriptional activators required for class II major histocompatibility complex (HLA‐II) gene transcription in pluripotent stem cells can achieve HLA gene inactivation [97], but results in iPSCs lacking class I MHC and being lysed by natural killer (NK) cells. Subsequently, researchers therefore developed two new strategies for HLA cloaking: one is to introduce a chimeric molecule consisting of HLA‐E and B2M [98], or to delete both HLA‐A alleles, both HLA‐B alleles, and one HLA‐C allele, leaving one intact HLA‐C to avoid NK cell activation. The summary and comparison of the above two methods are as follows. The advantage of the HLA haplobank is that it does not require gene editing and retains natural immune surveillance function, thus offering higher safety, but its disadvantage is the need to establish a large number of cell lines resulting in high costs, and it still cannot completely avoid the risk of NK cell attack. The HLA cloaking strategy requires only a very few cell lines to cover the global population through gene editing, greatly reducing production costs and regulatory complexity. However, this strategy also comes with potential risks of gene editing and loses immune surveillance capability against cancer and infection due to alteration of MHC molecules.

In the construction and quality control of pluripotent stem cell (iPSC) banks, genomic sequencing strategies are undergoing a transition from whole‐exome sequencing (WES) to whole‐genome sequencing (WGS). Although WES effectively detects coding region variations, it covers only about 1%–2% of the genome sequence and cannot systematically evaluate functional variations in important regulatory regions such as promoters, enhancers, insulators, and non‐coding RNA genes [98]. Mutations in these non‐coding regions have been confirmed to be closely related to the maintenance of cell pluripotency, directional differentiation efficiency, post‐transplantation tumor risk, and immunogenicity regulation [99]. Relying solely on WES will miss such potential genetic defects. In contrast, WGS can comprehensively identify single nucleotide variations (SNVs), insertions and deletions (INDELs), structural variations (SVs), copy number variations (CNVs), and non‐coding variations, providing more complete genomic information, and is recommended as the gold standard for genetic testing in iPSC bank construction and cell therapy product development. Furthermore, the integration of single‐cell sequencing technology further improves the sensitivity of mutation detection, enabling the identification of somatic mutations at the clonal level and the separation of non‐mutated cell populations with the help of high‐precision monoclonal screening systems, such as VIPS single‐cell plater and CMX monoclonal imager, thereby controlling the accumulation of genetic load at the source.

In summary, exploring and solving the genomic impact of allogeneic iPSCs, achieving HLA matching optimization, genetic load minimization [100], and epigenetic stability control, building higher quality and more reliable stem cell biobanks, will promote the entire field of regenerative medicine to develop in a more responsible and sustainable direction, ultimately benefiting patients.

## 3 Applications of iPSCs in ALS

iPSCs' remarkable versatility establishes them as a critical platform for disease modeling, regenerative medicine, and drug discovery. In neurodegenerative diseases such as ALS, iPSCs and their derivatives demonstrate significant promise for overcoming challenges in both research and therapeutic development [101, 102].

### 3.1 Advancing Neurodegenerative Disease Research With iPSCs

In neurodegenerative disease research, mouse models and stem cell‐derived models are widely used to elucidate numerous pathogenic molecular mechanisms underlying these disorders. However, drugs identified through screening in these models often fail to translate effectively to human therapies. Recently, increasing emphasis has been placed on evaluating the relevance of the pathological mechanisms and effective treatments discovered in mouse models for human applications. This can be attributed to two key factors: the genetic differences between mice and humans, and the fact that many mouse models rely on the overexpression of pathogenic genes to mimic the disease, which fails to fully replicate human conditions. While studies have shown that overexpression of these genes in mice can induce corresponding pathological phenotypes, drugs identified through screening in these models often do not translate effectively to human clinical settings. This underscores the limitations of such models in accurately predicting therapeutic efficacy for human diseases [103, 104, 105, 106, 107].

Compared to mouse models, iPSC‐derived models, such as iPSC‐derived motor neuron models, iPSC‐derived cortical neuron models, and iPSC‐derived brain organoids, have increasingly attracted the attention in neurodegenerative diseases. The construction of these models primarily involves two approaches. The first is the generation of patient‐specific iPSCs, which are then differentiated into relevant neuronal cell types for studying disease mechanisms and conducting drug screening. The second approach involves the use of gene‐editing technologies, such as CRISPR/Cas9 or TALENs, to introduce pathogenic mutations into iPSCs, which are subsequently differentiated for disease modeling and further research. The advantage of the first approach lies in its ability to generate patient‐specific iPSC‐based disease models encompassing individuals' complete genetic profiles. This is particularly valuable for studying diseases with predominantly sporadic etiologies, such as amyotrophic lateral sclerosis (ALS). By utilizing iPSCs derived from multiple patients, this method allows for more comprehensive investigations into pathogenic mechanisms and aids in the identification of drugs that may be effective across diverse patient populations. The combination of iPSC technology and gene editing facilitates the creation of disease models with well‐defined or suspected pathogenic mutations, essential for elucidating the mechanisms of specific disease genes. Additionally, when patient samples are unavailable or patient‐specific iPSCs cannot be generated, gene editing allows for the development of iPSC models with targeted mutations for further investigation. The integration of patient‐derived somatic cell reprogramming with gene editing technologies holds significant potential for advancing iPSC‐based human disease models in research [108, 109, 110]. In the research and application of iPSCs and their derivatives in neurodegenerative disease treatment, the primary focus is on the use of the iPSC‐derived motor neuron (iPSC‐MN) model in the study of ALS.

### 3.2 Applications of iPSC‐MN in ALS

ALS is characterized by the progressive and irreversible degeneration of both upper and lower motor neurons. Early symptoms include muscle weakness, atrophy, fasciculations, and stiffness, which gradually progress to generalized muscle paralysis, ultimately leading to respiratory failure and death. The exact pathogenesis of ALS remains unclear, although it is believed to involve abnormalities in protein homeostasis, axonal transport, the cytoskeleton, and mitochondrial function. Currently, treatment options for ALS are limited, with only a few drugs, such as Riluzole and Edaravone, showing modest efficacy in slowing disease progression. However, these treatments provide benefits to only a small proportion of patients. The global incidence of ALS is approximately 1.5 to 2.5 cases per 100,000 people annually. It is a highly heterogeneous, rare disease, with around 90%–95% of cases being sporadic and only 5%–10% familial. More than 50 genes have been identified as associated with ALS, including SOD1, C9orf72, UBQLN2, TDP‐43, CHCHD10, hnRNPA1, KIF5A, and OPTN, among others [111, 112, 113]. Consequently, iPSC‐MNs offer a promising platform for investigating ALS pathogenesis and screening potential drugs.

iPSC‐MNs from ALS patient‐specific lines often effectively replicate disease‐related pathological features, including neurofilament fragmentation, tangling, and persistent neuronal death. In fully differentiated mature iPSC‐MNs, the aberrant cytoplasmic localization of TDP‐43 and the formation of protein aggregates can be observed, closely resembling the clinical features of ALS [114, 115, 116]. Currently, various small molecule combinations have been developed to direct the differentiation of iPSCs into motor neurons. These approaches predominantly rely on dual SMAD inhibition, which involves the suppression of bone morphogenetic proteins (BMP) and transforming growth factor β (TGF‐β) signaling pathways to recapitulate the formation of the neural ectoderm during embryonic development. Li et al. later demonstrated that sustained activation of the WNT signaling pathway, when combined with dual SMAD inhibition, more efficiently drives the differentiation of iPSCs into neural epithelial progenitor cells and neural progenitor cells. Following the generation of neural progenitors, treatment with retinoic acid (RA) and SHH agonists, such as Purmorphamine and Smoothened Agonist (SAG), further promoted their differentiation into motor neurons. The addition of Notch signaling inhibitors supported the generation of functional, mature motor neurons. In ALS patient‐derived motor neurons, including neurotrophic factors such as BDNF and GDNF helped somewhat mitigate neuronal cell death [117, 118, 119, 120, 121].

Using iPSC‐MN models, researchers have identified potential therapeutic drugs for ALS, including Retigabine, Bosutinib, and Ropinirole [122, 123, 124, 125, 126, 127]. Retigabine, a KCNQ2/3 voltage‐gated potassium channel opener, alleviates glutamate‐induced excitotoxicity and was initially developed for treating epilepsy. In ALS, motor neuron hyperexcitability plays a critical role in neuronal degeneration. Wainger et al. established iPSC‐MN models carrying SOD1, C9orf72, and FUS mutations and found that Retigabine effectively suppressed the hyperexcitability of differentiated motor neurons. Clinical trials have further demonstrated that Retigabine has shown efficacy in treating several ALS patients, although it does not provide a cure [123]. Bosutinib is a tyrosine kinase inhibitor used in the treatment of chronic myelogenous leukemia, where it effectively suppresses tyrosine kinase activity. Imamura et al. found that several small molecules inhibiting tyrosine kinase activity can prevent the sustained death of neurons derived from ALS patient iPSCs. Notably, Bosutinib facilitates the autophagic clearance of misfolded SOD1 toxic proteins and significantly protects neurons with TDP‐43 mutations or C9orf72 repeat expansions. In vivo, Bosutinib extended the lifespan of SOD1 mutant ALS mice. While clinical trials have shown a favorable safety profile, further investigation into potential dose‐dependent toxicity remains necessary [124]. Through screening in iPSC‐MNs derived from sporadic ALS patients, Koki Fujimori et al. identified Ropinirole, which shows promising therapeutic effects. It effectively targets key ALS‐related pathologies, including the mislocalization of FUS and TDP‐43, abnormal aggregation of stress granules, and neuronal fragmentation [128]. Ropinirole, initially developed for the treatment of Parkinson's disease, is a dopamine D2 receptor (D2R) agonist. It exerts neuroprotective effects by coupling with G‐inhibitory proteins (Gi), which inhibit adenylate cyclase activity, reducing cyclic adenosine monophosphate (cAMP) production and alleviating neuronal hyperexcitability. They also discovered that Ropinirole initiates a neuroprotective mechanism independent of the D2R pathway, enhancing mitochondrial function and conferring resistance to oxidative stress and abnormal protein aggregation [129]. Subsequent clinical trials have demonstrated that Ropinirole exhibits good safety and tolerability across multiple ALS patient cohorts, effectively slowing disease progression. Larger‐scale clinical studies are currently underway. These findings underscore the effectiveness of iPSC‐MNs in studying the pathogenesis of ALS and in screening for potential therapeutic agents.

While iPSC‐MN models induced by small molecule combinations have effectively recapitulated various ALS pathologies and facilitated the screening of potential therapeutic compounds, significant challenges remain. One major limitation lies in their predominant focus on lower motor neurons, raising critical questions about their ability to represent upper motor neurons. Specifically, it remains uncertain whether ALS‐related pathological features observed in spinal motor neurons are replicated in cortical neurons, or whether therapeutic compounds identified through iPSC‐MNs are equally effective in targeting upper motor neurons [130]. Moreover, key challenges in induction technologies include obtaining single‐cell motor neurons and developing more efficient protocols. How to isolate single‐cell motor neurons is crucial for studying protein expression, distribution, and quantifying both normal and aberrant neurons [131]. Studies have shown that dissociating neuronal clusters at the motor neuron stage can yield single‐cell motor neurons. However, this process inevitably causes significant neuronal damage, as motor neurons typically have long axons at this stage. The resulting morphological abnormalities, such as axonal breakage caused by dissociation, may be misinterpreted as pathological features of ALS. Recent studies indicate that single‐cell dissociation of neuronal clusters followed by differentiation at the neural progenitor stage may be an effective strategy. At this stage, neural progenitors have already undergone directed differentiation, and the axons are relatively short, minimizing the risk of significant damage during dissociation. However, motor neurons derived from this approach still require comprehensive functional testing [132, 133]. Table 2 compares the differences between dissociating neuronal clusters at the motor neuron stage to obtain single‐cell motor neurons and direct induction of fibroblasts to induced motor neurons (iMNs). The iPSC‐MN model has proven highly valuable in ALS pathogenesis research and drug screening. Clinical trials using iPSC‐derived products for ALS began as early as 2014 [134]. With ongoing technological advancements, safer, more stable, and higher‐quality iPSC‐derived products are expected to enhance ALS therapy.

**TABLE 2:** Comparison between classical iPSC‐MN reprogramming and directly induced iMNs strategies.

Key Parameter	Classical iPSC‐MN Differentiation	Direct iMN Reprogramming
Intermediatestages	Pluripotent → Neural Progenitors	No pluripotent stage
Total duration	8–12 weeks	3–4 weeks
Teratoma risk	Requires stringent elimination	None
Epigenetic memory	May retain fibroblast signatures	Maintains donor epigenome
Clinical readiness	Established GMP protocols	Under process development
Standardization	QC standards implemented	Lacking unified criteria

## 4 Discussion

Several limitations persist in the use of patient‐derived iPSC models. Firstly, approximately 95% of ALS cases are sporadic, with etiology involving complex interactions between genetic and environmental factors [135]. Therefore, when using iPSCs derived via classical reprogramming methods for therapeutic purposes, several challenges arise beyond the well‐known risk of teratoma formation. While autologous iPSCs can largely preserve the patient's native epigenetic landscape [93], they suffer from prolonged differentiation protocols, high costs, and substantial time investments—potentially delaying optimal treatment windows [134]. On the other hand, allogeneic iPSCs lack the patient‐specific epigenomic imprint [136], which may affect their relevance and safety. Moreover, prior to clinical application, it is essential to perform WGS of donor‐derived iPSC lines to evaluate additional pathogenic or likely pathogenic variants, as well as potential adverse effects linked to non‐coding region variations [101, 137].

Since its inception, iPSC technology has undergone remarkable development. Continuous optimization of reprogramming methods and culture conditions has significantly improved its efficiency, safety, and stability, making it invaluable for both basic research and clinical applications. iPSC‐derived modelshave played a transformative role in advancing disease modeling and uncovering pathogenic mechanisms in complex neurodegenerative disorders. Furthermore, neural progenitor cell derivatives have demonstrated substantial therapeutic potential in clinical contexts [127, 138, 139, 140, 141]. Despite significant advancements, iPSC technology continues to face considerable challenges in clinical translation and large‐scale application, particularly regarding the quality, stability, and safety of derived cell products. Addressing issues such as the “uniformity” and standardized quality of iPSC‐derived products requires further refinement in current research and regulatory frameworks. Under these circumstances, chemical reprogramming, characterized by its easily standardized processes, emerges as a promising alternative that may help overcome these limitations.

iPSC technology, established less than two decades ago, is at a pivotal stage in transitioning from basic research to clinical application. Before it can be fully implemented in clinical settings, it is essential to establish rigorous quality standards, regulatory frameworks, and comprehensive guidelines. Equally important is the need for a deeper understanding of iPSCs. Although iPSCs and their derivatives have shown considerable promise in aesthetic medicine, anti‐aging, cancer therapy, and neurodegenerative disease treatments, they should not be viewed as a panacea. There are still significant uncertainties surrounding their application, and unrealistic expectations could lead to the misuse of this technology. Overcoming the remaining challenges will open the door to broader clinical applications, positioning iPSC technology as a transformative tool in disease research and therapy, with the potential to revolutionize human health globally.

## Author Contributions

Conceptualization, supervision, and funding acquisition, Z.X. and Z.L.; writing – original draft and visualization, Y.L; writing – review and editing, Z.X. and Y.L. All authors have read and agreed to the published version of the manuscript.

## Disclosure

Disclaimer: The statements, opinions and data contained in all publications are solely those of the individual author(s) and contributor(s) and not of MDPI and/or the editor(s). MDPI and/or the editor(s) disclaim responsibility for any injury to people or property resulting from any ideas, methods, instructions or products referred to in the content.

## Conflicts of Interest

The authors declare no conflicts of interest.

## Data Availability

No new data were created or analyzed in this study. Data sharing is not applicable to this article.
